# Implementation of outpatient schema therapy for borderline personality disorder: study design

**DOI:** 10.1186/1471-244X-9-64

**Published:** 2009-10-06

**Authors:** Marjon Nadort, Arnoud Arntz, Johannes H Smit, Josephine Giesen-Bloo, Merijn Eikelenboom, Philip Spinhoven, Thea van Asselt, Michel Wensing, Richard van Dyck

**Affiliations:** 1 GGZinGeest, Department of Psychiatry and Institute for Research in Extramural Medicine, VU University Medical Center Amsterdam, The Netherlands; 2 Maastricht University, Department of Clinical Psychological Science, The Netherlands; 3 Radboud University Nijmegen Medical Centre, Scientific Institute for Quality of Healthcare, The Netherlands; 4 Leiden University, Institute of Psychology and Department of Psychiatry, The Netherlands; 5 Maastricht University Medical Center, Department of Clinical Epidemiology and Medical Technology Assessment, The Netherlands

## Abstract

**Background:**

Schema Therapy (ST) is an integrative psychotherapy based upon a cognitive schema model which aims at identifying and changing dysfunctional schemas and modes through cognitive, experiential and behavioral pathways. It is specifically developed for patients with personality disorders. Its effectiveness and efficiency have been demonstrated in a few randomized controlled trials, but ST has not been evaluated in regular mental healthcare settings. This paper describes the study protocol of a multisite randomized 2-group design, aimed at evaluating the implementation of outpatient schema therapy for patients with borderline personality disorder (BPD) in regular mental healthcare and at determining the added value of therapist telephone availability outside office hours in case of crisis.

**Methods/Design:**

Patient outcome measures will be assessed with a semi-structured interview and self-report measures on BPD, therapeutic alliance, quality of life, costs and general psychopathology at baseline, 6, 12, 18 and 36 months. Intention-to-treat analyses will be executed with survival analysis for dichotomous variables, and one-sample t-tests and ANCOVAs for continuous variables with baseline as covariate and condition as between group factor. All tests will be two-tailed with a significance level of 5%.

**Discussion:**

The study will provide an answer to the question whether ST can be effectively implemented and whether phone support by the therapist has an additional value.

**Trial Registration:**

The Dutch Cochrane Center, NTR (TC = 1781).

## Background

Borderline Personality Disorder (BPD) is a disabling psychiatric disorder, which is characterized by substantial distress and disruptions in functioning. It has for long been viewed as a severe and difficult to treat psychiatric condition. However, during recent years several promising treatment possibilities have been developed. Among them, Schema Therapy (ST) was found to be effective regarding all aspects of BPD [1,2]. How well ST can be delivered in regular mental healthcare practice is unknown, but it is expected that its implementation poses challenges.

BPD is marked by chronic instability in multiple areas (emotional dysregulation, self-harm, impulsivity and identity disturbance). The lifetime prevalence of BPD in the general population is 1-2%. In psychiatric outpatient settings 10% of the patients suffer from BPD, in psychiatric inpatients settings 20% [3]. The medical and societal costs for BPD are substantial [2,4,5]. About 10% of the BPD patients die because of suicide [6,7].

However, recent years showed progress in the development of treatment options [8-14] that are supported by randomized controlled trials [1,7,15-19]. These treatments demonstrated effectiveness on symptom level, as manifested by reduced suicide attempts, fewer acts of self-harm or hospitalizations. Although pharmacological treatment can reduce symptoms, a Cochrane review indicates that there is no convincing evidence that any medication has complete success [20]. Psychotherapy is the necessary and primary treatment modality for BPD [21]. In a RCT which compared Schema Therapy and Transference Focused Psychotherapy (TFP) [1] both therapies showed a significant change in patients' personality, also at 1-year follow up [22]. This study showed that three years of ST and TFP proved to bring about a significant change in patient's personality, shown by reductions in all BPD symptoms and general psychopathologic dysfunction, increases in quality of life, and changes in associated personality features. While both treatment conditions showed positive results in the treatment of many aspects of BPD, ST was superior to TFP with respect to reduction in BPD manifestations, general psychopathologic dysfunction, and change in ST/TFP personality concepts. ST had a recovery rate of 45.5% and a reliable change rate of 65.9% at three years, whereas the dropout rate for ST (27%) was significantly lower than for TFP (51%). As a result of these findings, ST is considered as an evidence based treatment option for borderline personality disorder in the Multidisciplinary Dutch Guidelines on Personality Disorders [23].

Based on these positive results, a study of the implementation of ST in regular mental healthcare practice was planned. The rationale is that clinical interventions with proven effectiveness are not necessarily implemented in regular practice and, if implemented, treatment outcomes are not always equally good as in the clinical trial. One of the premises in the therapeutic approach of ST [11,13,14] and Dialectical Behavior Therapy [8,9,17,18] is that borderline patients need extra support of the therapist in between sessions when they are in crisis or in emotional need. For this reason patients are offered a special phone number where they can reach their therapist outside office hours. This personal connection between sessions is suggested to help to refute the patient's beliefs that there is nobody who really cares and can help to prevent or overcome crisis. In a pilot study of ST crisis support in the form of therapist phone accessibility outside office hours was one of the most controversial topics [24] and led some therapists to withdraw from the project. In general mental healthcare there is much discussion about this topic because of the financial consequences, the burden to and responsibility of the therapist, and the possible risk of violation of boundaries. Therefore, telephone accessibility outside office hours was perceived as an important barrier for the successful implementation of ST in regular practice. The RCT by Giesen-Bloo et al. [1] demonstrated that ST is a successful treatment, but it remains unknown whether the crisis support by the therapist was crucial to outcomes. Since the issue of crisis support outside office hours by the therapist makes it difficult to implement ST in regular practice and its effect has never been examined, we decided to investigate the role of the crisis support outside office hours in the implementation study by randomly allocating the crisis support outside office hours to 50% of the therapists.

In sum, this study will test the implementation of ST for BPD in regular mental healthcare and will compare two modalities: one with extra crisis support by the therapist outside office hours and one without such telephone support. The study has three aims. First, to assess whether patient outcomes after 1.5 years of ST will be the same when implemented in regular practice, compared to what was found in the RCT [1]. Since rigorous evaluations such as RCTs always imply controlled conditions, it is unclear to what extent their positive effects can be generalized to regular clinical practice. Treatment effects may be more modest outside RCTs because of different circumstances [25-27]. The second aim will be to assess the added value of therapist telephone availability outside office hours in case of crisis (TTA) during the 1.5 yrs of ST. The third aim will be to assess the problems that may arise during the implementation process.

## Methods/Design

### Study Design

This study is a multicenter randomized two-group trial for studying the added value of therapists phone support outside office hours. It is also a clinical evaluation of implementing ST for BPD and a comparison of the regular mental healthcare treatment results with those in a randomized clinical trial in academic settings. The interventions and assessments will be executed between December 2005 and August 2010.

### Recruitment/Settings and locations

#### Mental healthcare centers

Different mental healthcare centers, covering urbanized areas and located in various parts of the Netherlands will be approached and invited to take part in the implementation study. Selection criteria are a) at least two therapists on each location so that peer supervision groups can be formed, b) therapists agree in executing the telephone availability outside office hours and managers have to give their permission to do so, c) both therapists and managers have to agree in making the necessary time reservations for monthly supervision and weekly peer supervision.

#### Patients and procedures

Patients will be recruited within the departments of the mental healthcare centers. They can also be referred by therapists of other mental health institutes, primary care physicians or psychotherapists with private practices. Patients have to be referred based on a clinical diagnosis of BPD. At each site patients will be screened on the inclusion-exclusion criteria by specialized trained research assistants and be informed about the study. A positive screening procedure takes two months, and this interval serves as a patient's motivational check for undergoing intensive psychotherapy.

**Figure 1:**
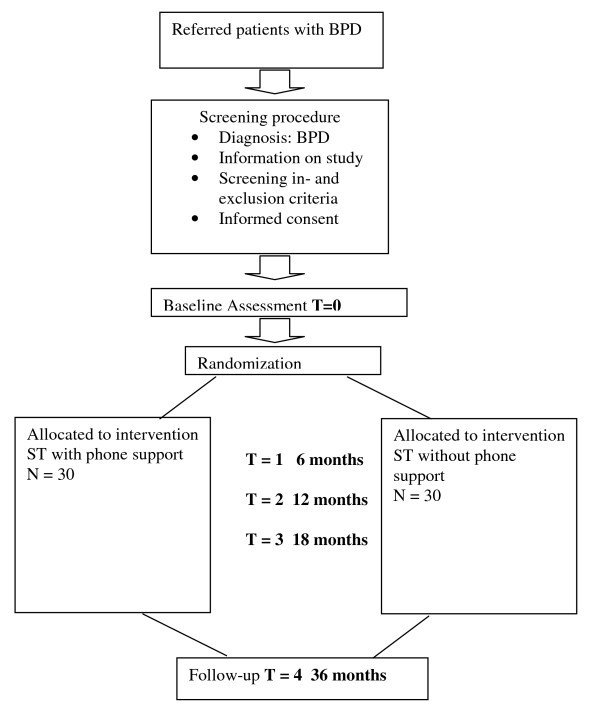
**Shows the procedures in a flow chart**.

If patients are willing and eligible to participate signed informed consent will be obtained after full explanation of the procedures and both conditions of ST at the first assessment and before randomization. See Figure 1 for the flowchart. Participants do not receive compensation for screening or assessments. Participating in assessments is obligatory to receiving the studied treatments.

### Participants

#### Inclusion criteria

Patients (aged 18-60) are eligible to participate if their main diagnosis is a Borderline Personality Disorder according to the DSM-IV criteria [3]. The Structured Clinical Interview for the Diagnostic and Statistical Manual of Mental Disorders, Fourth Edition (SCID-II) [28,29] will be used for assessing the diagnosis BPD. In addition, the level of symptom severity should be ≥ 20 on the Borderline Personality Disorder Severity Index (BPDSI-IV) [30,31]. Co morbid axis-I and axis-II disorders are allowed as is medication use.

#### Exclusion criteria

Patients are excluded from the study if they suffer from one or more of the following disorders: a psychotic disorder (except short, reactive psychotic episodes), bipolar disorder, dissociative identity disorder, antisocial personality disorder, attention deficit hyperactivity disorder, addiction of such severity that clinical detoxification is indicated (after which entering treatment is possible), psychiatric disorders secondary to medical conditions and mental retardation or if they do not have sufficient command of the Dutch language necessary to participate in the study.

#### Randomization

To prevent regional influences and enhance implementation TTA has to be equally spread over the different sites. Therefore a stratified randomization procedure will be used. The stratification procedure will be performed by a study-independent person and will be concealed for participating therapists, patients and researchers. Stratified per center, 50% of the therapists will be randomly allocated to the condition with extra phone support and 50% of the therapists to the condition without extra phone support. Each therapist will treat two patients either with or without phone support dependent upon the randomization. After completing the baseline assessment and signing the informed consent form patients will be randomly assigned to one of therapists of the participating institutes in their regions.

#### Assessments

Data are collected at four points in time: at baseline (T0), after 6 months of treatment (T1), 12 months of treatment (T2) eighteen months of treatment (T3) and a three- year follow-up (T4). Experienced research assistants with higher vocational training in psychology will be trained on the different sites in assessing patients for treatment outcome measures. Study researchers, screeners, research assistants and therapists are masked to treatment allocation during the screening period and the first assessment.

Table 1 summarizes the measures that are used at each point.

**Table 1:** Summary of measures

**Measure**	**Baseline**	**6 months**	**12 months**	**18 months**	**Follow-up 36 months**
**Interview:**					

Mini International Neuropsychiatric Interview (MINI)	x				

Demographics	x				

Structured Clinical Interview for DSM-IV, axis II (SCID-II), section Borderline Personality Disorder	x				

Borderline Personality Disorder Severity Index-IV (BPDSI-IV)	x	x	x	x	x

(Dutch Adult Reading Test)	x				

					

**Self report measures:**					

Borderline PersonalityDisorder-47 (BPD-47)	x	x	x	x	x

Symptom Check List 90 (SCL-90)	x	x	x	x	x

European Quality of Life (EuroQol)	x	x	x	x	x

World Health Organization Quality of Life (WHOQol)	x	x	x	x	x

Young Schema Questionnaire L 2 (YSQ)	x	x	x	x	x

					

**Questionnaire for economic evaluation:**					

Cost interview	x	x	x	x	x

					

**Questionnaires for therapeutic relationship**:					

Working Alliance Inventory, patient version (WAI-P)	x	x	x	x	x

					

**Questionnaire filled in by therapists:**					

Working Alliance Inventory, therapist version (WAI-T)	x	x	x	x	x

Difficult Doctor Patient Relationship Questionnaire (DDPRQ)	x	x	x	x	x

The M.I.N.I. [32,33] will be used for assessing the Axis I diagnosis. The BPD section of the Structured Clinical Interview for the Diagnostic and Statistical Manual of Mental Disorders, Fourth Edition (DSM-IV) (SCID-II) [28,29] will be used for assessing the diagnosis BPD. If Antisocial Personality Disorder is suspected patients will not be included. To assess the severity of the borderline complaints patients will be screened using a semi structured clinical interview, the Borderline Personality Disorder Severity Index, fourth version (BPDSI-IV; range 0-90) [30,31]. A BPDSI-IV cut off score of ≥ 20 discriminates patients with BPD from patients with other personality disorders [31]. Further, if illiteracy is suspected, the Dutch Adult Reading Test [34] will be administered.

### Outcome measures

#### Primary outcome measure

The primary outcome measure is the score on the BPDSI-IV, a DSM-IV BPD criteria- based semi- structured interview: this 70- item index represents the current severity and frequency of the DSM-IV BPD manifestations. This instrument shows excellent psychometric features (Cronbach's alpha = 0.85, interrater reliability, 0.99; validity and sensitivity to change [30,31]. Previous research [30,31] found a cut-off score [35] of 15 between patients with BPD and controls, with a specificity of 0.97 and a sensitivity of 1.00.

#### Recovery criterion

The recovery criterion is, therefore, defined as achieving a BPDSI-IV score of less than 15 and maintaining this score until the last assessment.

#### Reliable change

A second criterion is reliable change [35], which reflects individual clinically significant improvement. For the BPDSI-IV, reliable change is achieved when improvement is at least 11.70 points at the last assessment [22].

#### Secondary outcome measures

##### EuroQol and WHOQol

Information on demographic factors (age, gender, marital status, education and employment status) will be collected at baseline. A secondary outcome measure is quality of life, which will be assessed by means of two widely used and psychometrically sound self-report questionnaires: the EuroQol-thermometer and EQ-5D and the World Health Organisation Quality of Life Questionnaire [36-39]. The vertical EuroQol-thermometer rating indicates one's experienced level between worst (0) and best (100) imaginable health status. The EQ-5D contains 5 dimensions: mobility, self care, daily activities, pain/discomfort and depression/anxiety. Each dimension is rated at three levels: no problems, some problems and major problems. EQ-5D health states can be converted into utility scores ranging between -0.59 and 1, with higher utility scores representing a better quality of life. The WHOQOL is a 100-item self-report questionnaire, and through the domains of physical health, psychological health, environment, personal convictions, social relationships and extent of independency, the WHO concept of quality of life is assessed.

### BPD-47, SCL-90, Young Schema Questionnaire

Other secondary outcome measures are measures for general psychopathologic dysfunction and measures of ST personality concepts, all in self-report format and with robust psychometric properties. These measures include the BPD Checklist on the burden of BPD-specific symptoms [40] and the Symptom Checklist-90 for subjective experience of general psychopathology [41,42]. A theory specific instrument is the Young Schema Questionnaire on schemas underlying Young's theory [43-46].

#### Economic evaluation

In addition to the clinical evaluation, an economic evaluation will be performed to assess the cost-effectiveness of ST with versus ST without extra phone support outside office hours. In the cost-effectiveness analysis of ST with versus ST without phone support, the difference in costs will be related to the difference in effectiveness, resulting in an Incremental Cost Effectiveness Ratio (ICER). The cost-effectiveness analysis will be based on two different effectiveness outcomes. First, cost-effectiveness will be based on the proportion of patients recovered according to the BPDSI-IV, this reflecting the investment needed to cure one patient. Secondly, cost-effectiveness will be based on Quality Adjusted Life Years (QALY), which are calculated with the EQ-5D utility scores, resulting in costs per QALY. The cost-effectiveness analysis will be based on the principles of a societal perspective using a time horizon of 18 months. Costs will be monitored by means of a cost-interview that will take place during the patient interview alongside the other measurements. The cost-interview contains items about paid and unpaid work, study, daily activities, family burden, paid help, use of healthcare and social services, use of medication, consumption of alcohol and drugs and out-of-pocket expenses. Also the number of face-to-face and telephone contacts with the study therapists will be registered.

#### Therapeutic Alliance

This study will also investigate the quality and the development of the therapeutic alliance as a mediator of change in ST. In the RCT [1] scores for the therapeutic alliance were higher in ST than in TFP. Negative ratings of therapists and patients at early treatment were predictive of dropout, while increasingly positive ratings of patient in the first half of treatment predicted subsequent clinical improvement [47]. Therapeutic alliance will be measured by the Working Alliance Inventory (WAI) and the Difficult Doctor-Patient Relationship Questionnaire - Ten Item Version (DDPRQ-10).

The WAI [48] is one of the most commonly used and extensively validated measure of the alliance. It has been found to predict therapy outcome in numerous studies [49,50]. The Dutch version of the WAI consists of three subscales of 12 items each, rated on a 5-point in stead of 7-point Likert-type scale ranging from 1 ("never") to 5 ("always"). The subscales based on Bordin's [51] working alliance theory address agreement about the goals of therapy, agreement about the tasks of therapy, and the bond between the client and therapist. Patients have to complete the patient form (WAI-P) measuring the contribution of the therapist to the alliance as perceived by the patient and therapists have to complete the therapist form (WAI-T) in which they rate the contribution of the patient to the alliance. Because of the high intercorrelations among subscales (WAI-P range: .69 - .88; WAI-T range: .67 - .89) subscale mean scores are added together to derive a global score. A higher score on the WAI indicates a higher quality of the working alliance.

Difficult Doctor-Patient Relationship Questionnaire - Ten Item Version (DDPRQ-10). The DDPRQ [52] is a self-report questionnaire, which aims to measure the extent to which patients are experienced as frustrating or difficult in the therapeutic relationship by their doctor or therapist and provoke levels of distress that transcend the expected and accepted level of difficulty. Of the DDPRQ-10 five items are about the therapist's subjective experience (e.g., "Do you find yourself secretly hoping that this patient will not return?"), four are quasi-objective questions about the patient's behavior (e.g., "How time consuming is caring for this patient?"), and one item about symptoms combines elements of the patient's behavior and the therapist's subjective response (i.e. "To what extent are you frustrated by this patient's vague complaints?"). The items are answered on a 6-point Likert-type scale ranging from 1 ("not at all") to 6 ("a great deal"). The total score of the DDPRQ equals the mean of the 10 items. A higher score indicates a higher level of therapist frustration.

#### Treatment Adherence

Treatment adherence will be monitored by means of supervision. All sessions will be audiotaped. The audiotapes will be saved for evaluation. Of all patients one audiotape between 5 and 12 months of treatment will be randomly selected. Twenty tapes will be rated by independent raters to assess the intra class correlation coefficient (ICC). The raters will be independent of the study and masked to treatment condition and outcome. The raters will be psychologists trained in ST. We will use the ST Therapy Adherence and Competence Scale for BPD [53]. This instrument consists of visual analogue scales and Likert scale items and has a competence cutoff score of at least 60.

#### Registration of the phone contact

All therapists of the condition with phone support outside office hours have to monitor the telephone contacts on standardized forms with the following specifications: duration of the contact (minutes), time (weekday/nights or weekend), point of time (day, evening, night), reason of the phone contact (crisis, therapeutic, administrative). All contacts will be registered and used for calculating the number of therapeutic and crisis contacts outside office hours. The data will also be used for another yet to publish cost-outcome article.

#### Registration of therapy sessions

Therapists have to monitor the number of sessions. The content of the sessions and the used ST-techniques have to be registered on standardized forms.

#### Problems during the implementation process

These will be monitored by the researcher, recorded in a log book, and discussed with the project group during monthly meetings and with the therapists during the monthly supervision. Possible topics that will be discussed are the experiences of therapists and research assistants with the project, no show or drop-outs of patients, support of therapists by management, peers, and crisis facilities, and organizational changes influencing the implementation process like reorganizations.

#### Implementation interventions

On the basis of explorations of possible facilitators and barriers, the following implementation interventions will be applied to enhance successful implementation [54]. Firstly, therapists, managers and assistants of different mental healthcare centers will be informed of the study. Secondly, agreements will be made with the therapists and managers about the time investment for the treatment protocol (sessions twice a week, peer supervision weekly and supervision once a month) and financial aspects. Thirdly, therapists and research assistants will be trained and support on organizational level will be offered. The process evaluation aims to assess the impact of these implementation interventions on the delivery of ST for BPD patients and to analyze the problems that may occur during the implementation process.

#### Training and supervision

As the primary aim of the study is to assess whether ST can be successfully implemented in regular mental healthcare practice, we will make the following adjustments compared to the Giesen-Bloo et al. trial. In the study of Giesen-Bloo et al. [1] the ST therapists were trained and supervised by the originator of ST, Jeffrey Young, in the implementation study the therapists will be trained and supervised by Dutch experts [55,56]. The training will be based on a structured and piloted program supported by a set of DVDs with examples of ST techniques, see Nadort et al. [54].

Therapists will be trained in a 50 hours training program (eight days during a period of two months). Essential to the treatment is expert supervision and peer supervision. During the first year monthly supervision will be provided on each site, in the second year supervision will be provided every two months. The therapists will have weekly peer supervision on each site. There will be a 1-day central supervision for all therapists once a year.

#### Frequency of sessions and treatment period

In the RCT [1] the treatment period was three years with sessions twice a week. In the implementation study there will be sessions twice a week in the first year, but sessions once a week in the second year. In the implementation study we decided to do the first evaluation after a treatment period of eighteen months. This was decided for several reasons: different treatments have shown positive results after 1-1,5 years of treatment [1,15,16,18], effectiveness already became apparent after one year [1] and most drop outs occurred during the first 1,5 years of therapy [1,15].

#### Treatment Protocol

Treatment will be offered in 45-minutes sessions twice a week in the first year and once a week in the second year. Treatment protocols address the theoretical model, treatment frame, different phases and the use of strategies and techniques [11,13,14,55-58]. Central to ST is the assumption of 5 schema modes specific for BPD. Schema modes are sets of schemas expressed in pervasive patterns of thinking, feeling and behaving [59,60]. Change is achieved through a range of behavioral, cognitive and experiential techniques that focus on (1) the therapeutic relationship, (2) daily life outside therapy and (3) past (traumatic) experiences. Recovery in ST is achieved when dysfunctional schemas no longer control or rule the patient's life.

## Sample size and Data Analysis

### Sample size

The BPDSI-IV power calculation is based on the aim of showing a difference at the patient level between the conditions with extra phone support of the therapist outside office hours versus the condition without such support. Because we do not know what the effect of the extra phone support is, it is decided to use a medium effect size of 0.5, according to Cohen [61], for the power calculation. With a minimum of 2 × 30 patients per condition, the power to demonstrate such a difference between the two conditions with two-tailed alpha of 0.05 is 84. Therefore a minimum of 60 patients is required and accordingly 30 therapists need to be recruited.

### Analysis

The statistical analyses will be based on the intention-to-treat as randomized principle. Treatment effects will be tested with survival analysis for dichotomous variables, and one-sample t-tests and ANCOVAs for continuous variables with baseline as covariate and condition as between group factor. When no deviations from distributional assumptions are detected, parametric ANCOVAs will be used.

Using Cohen's formula, effect sizes will be calculated as X_1_-X_2_/SD_pooled_, were X_1 _represents the pre-treatment scores, X_2 _the post-treatment scores, and SD_pooled _represents the pooled standard deviations of the pre- and post-treatment scores.

All the tests will be two-tailed with a significance level of 5%. Analyses will be performed using the Statistical Package for Social Sciences, version 15.0 for Windows (survival analyses, within-group analyses, Chi-square tests).

In the RCT [1] a pre to post treatment effect size difference of d = 1.24 (Cohen's d) was found on the main patient outcome measure BPDSI on 18 months. In the present study, the same treatment will be less intensive and executed in non-academic practice, so that a lower effectiveness can be expected. We therefore tentatively estimate the pre-post difference as d = 1.0.

It will be concluded that extra crisis support is definitely helpful if at the patient level a medium effect difference is found between the conditions with and without extra phone support. A possible small difference in effect will, although indicating that the extra support is helpful, probably not convince clinicians to implement this extra availability in their regular practice.

For the economic evaluation, analysis will also be performed according to intention-to-treat principle. The uncertainty around the cost-effectiveness and cost-utility ratios will be analyzed using bootstrapping techniques. Results of these bootstraps will be presented in cost-effectiveness planes

(showing all bootstrapped cost-effectiveness combinations) and cost-effectiveness acceptability curves (CEACs) which represent the probability that the intervention is cost-effective, given a certain threshold for the costs per unit of effect gained.

#### Ethical principles

The participation in the study is voluntary. Participants are informed that they can cancel their participation at any time without disclosing reasons for their cancellation and without negative consequences for their future care. Participants will sign an informed consent.

#### Vote of the ethics committee

The design and conduct of the study was approved by the Medical Ethics Committee for General Mental Healthcare (METIGG 5230).

## Discussion

This study protocol is presented here to offer researchers the opportunity to consider the methodological quality of this study with a critical view. Therapists can benefit by considering the information regarding the practical applications of the proposed protocol on borderline patients in secondary care. The number of studies on ST for BPD is small, while BPD affects a large group of patients in regular mental healthcare. Research into the effectiveness of ST when it is implemented in regular mental healthcare can make an important contribution to the improvement of care for BPD patients. Also research into the added value of phone support outside office hours provided by the therapist can make an important contribution to the application of ST.

### Strengths and limitations

Many methodological requirements for a high quality trial are met. Allocation is concealed through randomization by an external researcher. Recruitment of the patients will be done after the standard intake procedure at each site. As this study takes place within different general mental healthcare departments located in different parts of the Netherlands the results can, to a large extent, be generalized to the population of borderline patients seen in regular mental healthcare in the Netherlands. A limitation of the present study is that the assessments will be performed by research assistants who cannot remain blinded to the treatment condition of the included patients, as is always the case in trials studying the effects of psychotherapy. Nor are the patients blind to treatment condition. In this study, however, added to the main interview-based outcome measures, self-report questionnaires will be administered, that will not be influenced by the research assistants.

Another limitation is the power of this study. This implementation study is powered to demonstrate a medium or higher effect of TTA. The failure to detect any difference between conditions, does not mean that they are equivalent, only that differences, if any, will be small. A small difference however does not imply lack of clinical significance. But to detect a small effect with a significance level of .05 and a power of 80, a power analysis shows that a sample of more than 3100 patients is necessary, which is not feasible for a trial on long-lasting psychotherapy.

### Timeframe of the study

In December 2005 the randomized treatment study has been started up.

Month 1-6: Recruitment of departments, therapists and research assistants.

Month 6-8: Training of therapists and research-assistants.

Month 9-21: Screening and inclusion of borderline patients at the different locations. Start of the data collection for the treatment study. Supervision of the treatments. Start of data entry and purging of databases.

Month 27-43: Completion of data collection. Completion of purging and analysis of the data. Publication on the short-term findings.

Month 43 until 2010 Three-year follow-up assessments. Publication on the (cost-)effectiveness of ST. Dissemination of the results (e.g. presentation of study results at national and international conferences). Working on the implementation of ST in the Netherlands.

### Description of risks

There are no specific risks related to this study.

## Competing interests

The authors declare that they have no competing interests.

## Authors' contributions

MN, RvD, JS and AA developed the design of the randomized clinical trial and participated in writing the article. PS, MW, JG, ME, TvA advised on the content of the article. MN is the principal investigator and writer of this manuscript. All authors have read and approved the final version of the manuscript.

## Pre-publication history

The pre-publication history for this paper can be accessed here:


